# Sustained Na^+^/H^+^ Exchanger Activation Promotes Gliotransmitter Release from Reactive Hippocampal Astrocytes following Oxygen-Glucose Deprivation

**DOI:** 10.1371/journal.pone.0084294

**Published:** 2014-01-02

**Authors:** Pelin Cengiz, Douglas B. Kintner, Vishal Chanana, Hui Yuan, Erinc Akture, Pinar Kendigelen, Gulnaz Begum, Emin Fidan, Kutluay Uluc, Peter Ferrazzano, Dandan Sun

**Affiliations:** 1 Department of Pediatrics, University of Wisconsin School of Medicine and Public Health, Madison, Wisconsin, United States of America; 2 Department of Waisman Center, University of Wisconsin School of Medicine and Public Health, Madison, Wisconsin, United States of America; 3 Department of Neurological Surgery, University of Wisconsin School of Medicine and Public Health, Madison, Wisconsin, United States of America; 4 Department of Neurology, University of Pittsburgh, Pittsburgh, Pennsylvania, United States of America; 5 Department of Anesthesiology, Istanbul University, Cerrahpaşa Medical School, Turkey; University of South Florida, United States of America

## Abstract

Hypoxia ischemia (HI)-related brain injury is the major cause of long-term morbidity in neonates. One characteristic hallmark of neonatal HI is the development of reactive astrogliosis in the hippocampus. However, the impact of reactive astrogliosis in hippocampal damage after neonatal HI is not fully understood. In the current study, we investigated the role of Na^+^/H^+^ exchanger isoform 1 (NHE1) protein in mouse reactive hippocampal astrocyte function in an *in vitro* ischemia model (oxygen/glucose deprivation and reoxygenation, OGD/REOX). 2 h OGD significantly increased NHE1 protein expression and NHE1-mediated H^+^ efflux in hippocampal astrocytes. NHE1 activity remained stimulated during 1–5 h REOX and returned to the basal level at 24 h REOX. NHE1 activation in hippocampal astrocytes resulted in intracellular Na^+^ and Ca^2+^ overload. The latter was mediated by reversal of Na^+^/Ca^2+^ exchange. Hippocampal astrocytes also exhibited a robust release of gliotransmitters (glutamate and pro-inflammatory cytokines IL-6 and TNFα) during 1–24 h REOX. Interestingly, inhibition of NHE1 activity with its potent inhibitor HOE 642 not only reduced Na^+^ overload but also gliotransmitter release from hippocampal astrocytes. The noncompetitive excitatory amino acid transporter inhibitor TBOA showed a similar effect on blocking the glutamate release. Taken together, we concluded that NHE1 plays an essential role in maintaining H^+^ homeostasis in hippocampal astrocytes. Over-stimulation of NHE1 activity following *in vitro* ischemia disrupts Na^+^ and Ca^2+^ homeostasis, which reduces Na^+^-dependent glutamate uptake and promotes release of glutamate and cytokines from reactive astrocytes. Therefore, blocking sustained NHE1 activation in reactive astrocytes may provide neuroprotection following HI.

## Introduction

Hippocampal astrocytes are in intimate physical relationship with neurons. Each hippocampal astrocyte is in contact with several hundred dendrites from multiple neurons and envelope hundreds of thousands of synapses [1]. Thus, astrocytes play a direct role in hippocampal neuronal function by maintaining the ionic and neurotransmitter homeostasis of the synaptic interstitial space that is critical to synaptic transmission. Astrocytes can alter neuronal excitability through Ca^2+^ signaling or by release of synaptically active “gliotransmitters” (glutamate, ATP, and adenosine) [2]. Astrocytes can prevent neuronal energy failure by providing ATP during and after ischemia. In addition, glutamate uptake by glutamate transporters in astrocytes may decrease the detrimental effects of excessive glutamate release after ischemia [3]. Hippocampal astrocytes respond to neonatal hypoxia ischemia (HI) by developing reactive astrogliosis, which is characterized by up-regulation of glial fibrillary acid protein (GFAP) expression, astrocyte hypertrophy, and astrocyte proliferation [3], [4]. However, the significance of reactive astrogliosis and its contribution to hippocampal damage after HI in neonates is unknown.

Na^+^/H^+^ exchanger isoform 1 (NHE1) is a membrane protein that functions to regulate intracellular pH (pH_i_) by extrusion of one H^+^ in exchange for one Na^+^ [5]. Thus, acidosis following HI may trigger increased expression and activation of NHE1 to correct acidosis. We recently reported that expression of NHE1 protein was dramatically increased in hippocampal GFAP-positive reactive astrocytes at 72 h following HI in a neonatal mouse model [6]. We found that inhibition of NHE1 with its selective and potent inhibitor HOE 642 decreased CA1 pyramidal neuronal damage and improved motor and spatial learning [6]. We speculate that enhanced NHE1 activity in reactive astrocytes is detrimental and inhibition of NHE1 may offer neuroprotection in part via blocking NHE1 in reactive astrocytes. Here we report that NHE1 protein expression was up-regulated in hippocampal reactive astrocytes after *in vitro* ischemia (oxygen and glucose deprivation and reoxygenation, OGD/REOX). We also detected concurrent elevation of NHE1 activity, increased intracellular sodium concentration ([Na^+^]_i_) and intracellular calcium concentration ([Ca^2+^]_i_), and release of glutamate and cytokines from hippocampal astrocytes following OGD/REOX. Interestingly, inhibition of NHE1 activity significantly reduced all of these events. Therefore, we concluded that over-stimulation of NHE1 promotes gliotransmitter and cytokine release from reactive astrocytes, which can subsequently contribute to hippocampal neuronal damage under hypoxic and ischemic conditions.

## Experimental Procedures

### Materials

Hanks balanced salt solution (HBSS) and L-glutamine were from Mediatech Cellgro (Manassas, VA). BCECF-AM, fura2-AM, Sodium Green-AM, and the Amplex® Red Glutamic Acid/Glutamate Oxidase kit were from Invitrogen (Carlsbad, CA). Gramicidin, ionomycin, nigericin, calcein-AM, propidium iodide (PI) and L-leucine methyl ester were purchased from Sigma (St. Louis, MO). HOE 642 was a kind gift from Aventis Pharma (Frankfurt, Germany). DL-threo-b-benzyloxyaspartate (TBOA) was from Tocris (Ellisville, MO). Fetal bovine serum (FBS) was obtained from Valley Biomedical (Winchester, VA). Rabbit anti-NHE1 polyclonal antibody was purchased from Abcam (Cambridge, MA). ELISA kits for the cytokines IL-1, IL-6, and TNF-α were from R & D (Minneapolis, MN). Mouse anti-glial fibrillary acidic protein (GFAP) monoclonal antibody was from Cell Signaling (Danvers, MA). Monoclonal antibody cocktail against GFAP derived from the Bigner-Eng clones MAb1B4, MAb2E1 and MAb4A11 was from Convance (Princeton, NJ). Mouse anti-α tubulin monoclonal antibody was from Promega (Madison, WI). m.

### Primary Hippocampal Astrocyte Culture

This study was carried out in strict accordance with the recommendation in the Guide for the Care and Use of laboratory Animals of the National Institute of Health. The protocol was approved by the University of Wisconsin-Madison, Institutional Animal Care and Use Committee (Protocol G000614). Harvesting of tissue for culture was done under isoflurane anesthesia and all efforts were made to minimize pain. Dissociated hippocampal astrocyte cultures were prepared from the hippocampus of 3–4 day old mice as described before [7] with minor modifications. Hippocampal cortices were removed from the mice, minced, and rinsed in ice-cold HBSS. The cortices were then incubated in trypsin solution (0.25 mg/ml in HBSS) for 20 min at 37°C. Cells were then dissociated by gentle trituration in culture medium containing EMEM supplemented with 2 mM L-glutamine, 10% FBS, 100 IU/ml penicillin and 0.1-mg/ml streptomycin. Cells were then passed through a 40 µm cell strainer, centrifuged, and resuspended in culture media. Viable cells (4×10^4^ cells/6 well, 1×10^3^ cells/24 well) were either plated on glass coverslips (22×22 mm) coated with collagen type 1 placed in petri dishes, six well plastic culture plates, 24-well culture plates or 35 mm glass bottomed culture dishes (15 mm). Cultures were maintained in a 5% CO_2_ atmosphere at 37°C. To obtain morphologically differentiated astrocytes, confluent cultures (7 days in culture, DIV 7) were treated with EMEM containing 0.25 mM dibutyryl cyclic AMP (dBcAMP) to induce differentiation.

In some studies, three-day old astrocyte cultures were supplemented with 5 mM L-leucine methyl ester (LME) for 10 days to remove possible microglial contamination in the astrocyte cultures. LME is a microglial cytotoxic agent that has been used extensively as a method to eliminate proliferating microglia [8], [9]. After incubation, LME-treated cultures were subjected to a shaking protocol (300 RPM for 1 h) to remove possible contaminating microglia. Experiments were routinely performed in cultures 10–12 days old.

### OGD/REOX

Hippocampal astrocyte cultures were rinsed with an isotonic OGD solution (pH 7.4) containing (in mM): 0 glucose, 21 NaHCO_3_, 120 NaCl, 5.36 KCl, 0.33 Na_2_HPO_4_, 0.44 KH_2_PO_4_, 1.27 CaCl_2_, and 0.81 MgSO_4_. The cells were incubated in the OGD solution for 2 h in a hypoxic incubator (model 3130, Thermo Forma; Asheville NC) containing 94% N_2_, 1% O_2_, and 5% CO_2_. Normoxic control cells were incubated for 2 h in 5% CO_2_ and atmospheric air in a buffer identical to the OGD solution except for the addition of 5.5 mM glucose. For REOX, the OGD solution was replaced with EMEM containing 10% FBS and incubation at 37°C in 5% CO_2_ and atmospheric air.

### pH_i_ Measurement

pH_i_ measurement and prepulse treatment were performed as described previously [10]. Briefly, hippocampal astrocyte cultures grown on coverslips were incubated with 0.75–1.5 µM BCECF-AM at 37°C for 30 min. The coverslips were placed in a temperature controlled (37°C) open-bath imaging chamber (Model RC24, Warner Instruments, Hamden, CT) containing HCO_3_^–^free HEPES-MEM solution (mM, pH 7.4): 140 NaCl, 5.36 KCl, 0.81 MgSO_4_, 1.27 CaCl_2_, 0.44 KH_2_PO_4_, 0.33 Na_2_HPO_4_, 5.55 glucose and 20 HEPES. The chamber was mounted on the stage of the Nikon TI Eclipse inverted epifluorescence microscope and the cells visualized with a 40X objective. The cells were excited every 10–30 s at 440 and 490 nm, and the emission fluorescence at 535 nm recorded. Images were collected using a Princeton Instruments MicroMax CCD camera and analyzed with MetaFluor (Molecular Devices, Sunnyvale, CA) image-processing software. The ratio of the background-corrected fluorescence emissions (F490/F440) was calibrated using the high K^+^/nigericin technique [11].

For the pre-pulse treatment, cells were subjected to an acid load by a transient application (2.5 min) of a 30 mM NH_4_^+^/NH_3_ solution as described previously [10]. NH_4_^+^/NH_3_ solutions were prepared by replacing 30 mM NaCl in the HCO_3_^−^ -free, HEPES-buffered solution with an equimolar concentration of NH_4_Cl. pH_i_ recovery rate during the first min after NH_4_^+^/NH_3_ prepulse was determined from the slope of a fitted linear regression at ∼ pH 6.5 [12].

### [Ca^2+^]_i_ Determination

Hippocampal astrocytes grown on coverslips were incubated with 5 µM fura-2AM for 2 h at 37°C. The cells were rinsed with PBS and quickly (<2 min) placed on an open-bath imaging chamber and superfused (1 mL/min) with HCO_3_-MEM at 37°C. Using a TI Eclipse inverted epifluorescence microscope (40X objective), cells were excited every 10 sec at 340 and 380 nm, and the emission fluorescence at 510 nm was recorded. Images were collected and analyzed with the MetaFluor image-processing software. At the end of each experiment, the cells were exposed to 1 mM MnCl_2_ and 5 µM Br-A23187 in Ca^2+^-free HEPES-MEM. The Ca^2+^-insensitive fluorescence was subtracted from each wavelength before calculations [13]. The MnCl_2_-corrected 340/380 emission ratios were converted to [Ca^2+^]_i_ as described previously [13].

### Measurement of [Na^+^]_i_

[Na^+^]_i_ was determined using the fluorprobe sodium green-AM. Hippocampal astrocytes grown on glass-bottomed culture dishes (In Vitro Scientific, Sunnyvale, CA) were loaded with 15 µM sodium green-AM at 37°C for 2 h under normoxia, OGD, or REOX conditions. For OGD treatment, cells were incubated at 37°C with 200 µl OGD buffer for 2 h in a hypoxic incubator containing 94% N_2_, 1% O_2_, and 5% CO_2_. Normoxic control cells were incubated for 2 h in 5% CO_2_ and atmospheric air in a buffer (200 µl) identical to the OGD solution except for the addition of 5.5 mM glucose. Cells were then washed with HEPES-MEM and placed in a stage top incubator (Tokai Hit, Shizuoka-ken, Japan) at 37°C on the stage of Nikon A1 confocal microscope equipped with a perfect focus system and images collected every 20 sec (488 nm excitation, 525 emission) using a 60X objective. After a one min baseline collection, cells were subjected to an in-situ calibration using the ionophores gramicidin and monensin. Absolute [Na^+^]_i_ was determined as described before [14].

### Immunofluorescence Staining

Hippocampal astrocytes grown on coverslips were fixed in 4% paraformaldehyde in phosphate-buffered saline (PBS) for 15 min. After rinsing, cells were incubated with a blocking solution for 20 min followed by application of rabbit anti-NHE1 polyclonal antibody (1∶100) and mouse anti-GFAP monoclonal antibody (1∶500) diluted in the blocking buffer at 4°C overnight. After rinsing in PBS, cells were incubated with goat anti-mouse secondary antibody IgG (H+L) conjugated to Alexa Fluor® 488 (1∶200 dilution) and goat anti-rabbit secondary antibody IgG (H+L) conjugated to Alexa Fluor® 546 (1∶200 dilution) for 1 h at 37°C. Fluorescence images were captured with a Leica DMIRE2 inverted confocal laser-scanning microscope (40X objective).

### Immunoblotting

Hippocampal astrocyte cells were scraped from the flasks and lysed by 30 s sonication at 4°C in PBS (pH 7.4, 2 mM EDTA and protease inhibitors) [15]. Protein content was determined by the bicinchoninic acid method. Protein samples (60 µg/lane) and pre-stained molecular mass markers (Bio-Rad, Hercules, CA) were denatured in sodium dodecyl sulfate 5X sample buffer and then electrophoretically separated on 8% sodium dodecyl sulfate gels. After transferring to a nitrocellulose membrane, the blots were incubated in 5% nonfat dry milk in Tris-buffered saline for 2 h at room temperature, and then incubated with rabbit anti-NHE1 polyclonal antibody (1∶800) and mouse anti-GFAP monoclonal antibody (1∶1000) overnight at 4°C. After rinsing, the blots were incubated with horseradish peroxidase-conjugated secondary IgG at room temperature for 1.5 h. Bound antibody was visualized using an enhanced chemiluminescence assay (Amersham Corp., Burlington, MA).

### Glutamate Release Measurement

Glutamate released in cell culture medium was determined using an amplex® red glutamic acid/glutamate oxidase assay kit as per the manufacturer’s instructions. Hippocampal astrocytes (100,000 cells) grown on 20×20 mm coverslips in six-well plates were incubated with 200 µl OGD buffer for 2 h in a hypoxic incubator containing 94% N_2_, 1% O_2_, and 5% CO_2_. Normoxic control cells were incubated for 2 h in 5% CO_2_ and atmospheric air in the control buffer (200 µl). For medium sample collection at 1, 5 or 24 h REOX, 200 µl medium was collected and replaced with fresh medium. At the end of an experiment, 1 µl of medium was used for glutamate release measurement. Cells on the coverslips were lysed and protein content in each lysate was determined by the bicinchoninic acid method (BCA). The fluorescence (571 nm excitation/585 nm emission) of each medium sample was determined using a Gemini EM fluorescence microplate reader (Molecular Devices, Sunnyvale, CA). Glutamate concentration in medium sample (µM) was determined using a nine-point standard curve and all analyses were done in duplicate. The glutamate release in each sample was expressed as pg/µg lysate protein.

### Pro-inflammatory Cytokine Measurement

Hippocampal astrocytes cultured in six-well plates in medium (0.5 ml) were subjected to 2 h OGD plus 1, 5, or 24 h REOX. For analysis of TNF-α, IL-1β and IL-6 release in the medium, DuoSet cytokine ELISA kits (R&D systems, Minneapolis, MN) was used as per the instructions of the manufacturer. After removing the culture media for cytokine determination, the cells in each well were lysed and the protein concentration determined using the BCA method. 96-well plates were coated overnight with the respective diluted capture antibodies in 100 µl PBS/well (containing TNF-α, 14.4 µg; IL-1β, 72.0 µg; IL-6, 36.0 µg). After three washings with wash buffer (0.05% tween 20 in PBS, pH 7.2), plates were blocked (300 µl 1% BSA in PBS) for 1 h. Cytokine standards (100 µL) or culture medium samples (∼ 0.3 mg protein) were added and incubated at room temperature for 2 h. After the incubation, 100 µL of the biotinylated detection antibodies (TNF-α, 200 ng; IL-1β, 600 ng; or IL-6, 200 ng) were added to each well and incubated for 2 h. 100 µL of the diluted streptavidin-HRP (1∶200) antibody was added and plates were allowed to incubate for 20 min at room temperature. Following washing, chromogen was added and the reaction was stopped with a stop solution. The optical density of each well at 450 nm was determined immediately using a microplate reader (Molecular Devices, Sunnyvale, CA). A seven-point standard curve was generated with each run using supplied standards for each set of samples assayed. TNF-α, IL-1β or IL-6 was calculated with the standard curve and expressed as pg/mg protein. Based on the standard curve, the lowest detectable limits for TNF-α, IL-1β, or IL-6 were 31.25 pg/ml, 15.62 pg/ml, and 15.62 pg/ml, respectively.

### Cell Viability Assay

Hippocampal astrocytes grown on 24 well plates were subjected either to normoxia, 2 h OGD plus 5 h REOX, 5 h REOX +1 µM HOE 642, 24 h REOX or 24 h REOX +1 µM HOE 642. Cells were rinsed with HEPES-MEM and incubated for 30 min with 0.5 µM calcein-AM and 2 µg/ml PI in HEPES-MEM. Calcein (488 nm excitation, 525 emission) or PI (543 nm excitation, 610 emission) signals were obtained with a Nikon A1 confocal microscope under a 10X objective (Nikon Instruments, Melville, NY). Cell death was calculated by the ratio of PI positive cells to the number of calcein positive cells plus PI positive cells. The percent death calculated from each of 6 wells for per experimental condition was expressed as mean ± SEM.

## Statistical Analysis

Values are expressed as the mean ± SEM. Either a t-test or the Mann-Whitney rank sum test was used to compare the vehicle control and the drug-treated groups. ANOVA with Bonferroni post-test was used in the case of multiple comparisons. P-values smaller than or equal to 0.05 were considered statistically significant. Statistical analysis was performed using Sigmaplot (SPSS, Inc., Chicago, IL).

## Results

### Up-regulation of NHE1 Protein Expression in Hippocampal Astrocytes in *in vitro* Ischemia Model

**Figure 1:**
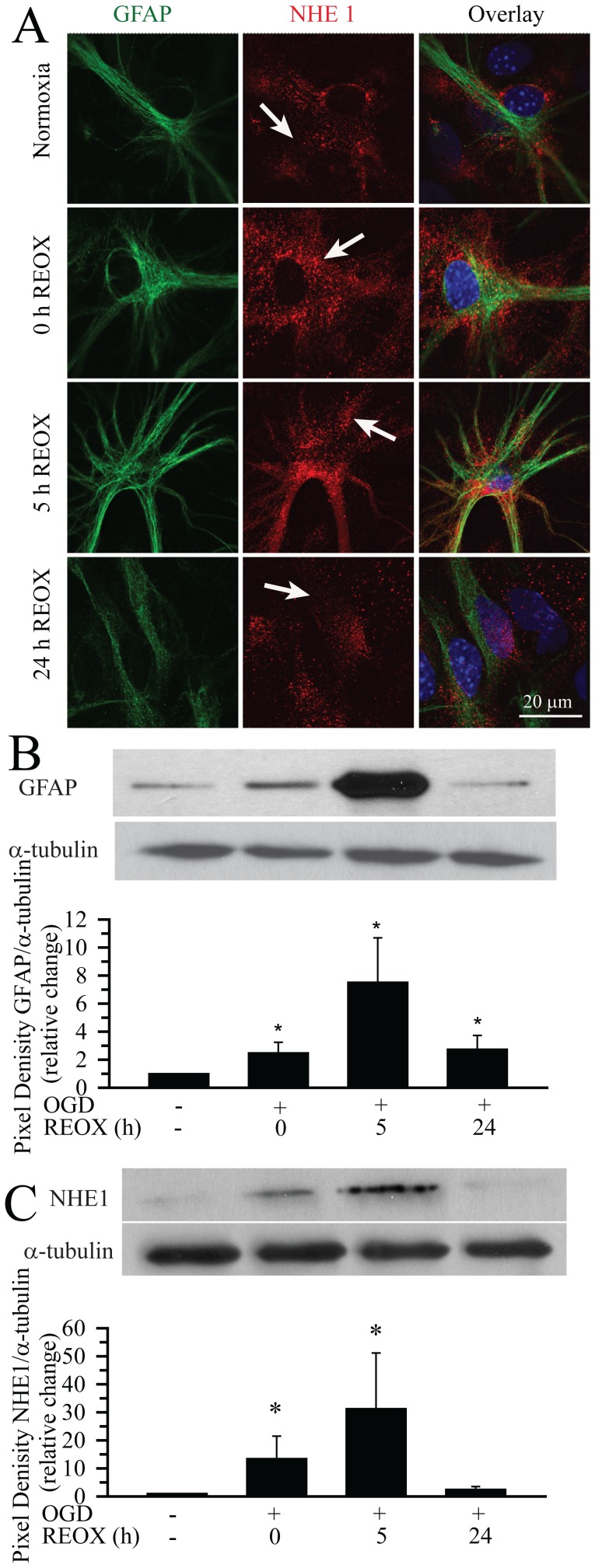
Up-regulation of NHE1 protein expression in reactive hippocampal astrocytes. **A.** Expression of NHE1 protein in hippocampal astrocytes. Immunofluorescence signals of NHE1 and GFAP were shown under normoxia control or OGD (2 h) plus 0 h, 5 h, or 24 h REOX. *Green*: GFAP, *red*: NHE1 (arrows), *blue*: To-pro-3 nuclear staining. Scale Bar: 20 µm. **B, C.** Quantification of GFAP (**B**) and NHE1 protein (**C**) expression with immunoblotting. Hippocampal astrocytes were subjected to 2 h OGD followed by 5 or 24 h REOX. Total pixel density in each protein band (minus background) was calculated using Image J. Data are expressed as the ratio of either GFAP or NHE1 to the corresponding α-tubulin band and normalized to the control. Data are mean ± SEM (n = 4). *p<0.05 vs. normoxia.

Basal level of NHE1 protein expression was detected in cultured hippocampal astrocytes under normoxic conditions (**Figure 1** **A, arrow**). NHE1 protein was expressed in all GFAP-positive astrocytes. At 2 h OGD as well as at 2 h OGD/5 h REOX, astrocytes exhibited classical reactive astrocytes morphology: retracted primary processes, hypertrophy of the soma and processes, and increased expression of GFAP. Interestingly, NHE1 immuoreactivity in hippocampal reactive astrocytes was enhanced at 2 h OGD/5 h REOX (**Figure 1** **A, arrow).** We further characterized the expression of GFAP and NHE1 following OGD by immunoblotting. Following 2 h OGD, GFAP expression was increased by ∼2.5-fold (**Figure 1** **B**). 2 h OGD/5 h REOX resulted in a further increase in GFAP expression to ∼ 7.5-fold of normoxic levels. Similar to the immunohistochemical staining data, GFAP expression returned toward normoxic levels after 24 h REOX. In the case of NHE1 expression, there was a 13-fold increase in NHE1 expression at the end of OGD, followed by an elevation to 30-fold of normoxic levels at 5 REOX (**Figure 1** **C**, bottom panel). At 24 h REOX, NHE1 expression nearly returned to normoxic levels. These results demonstrate that OGD/REOX triggered transformation of hippocampal astrocytes to reactive astrocytes. Expression of NHE1 protein was significantly increased in reactive astrocytes.

### Increased NHE1 Activity in Hippocampal Astrocytes Following OGD/REOX

**Figure 2:**
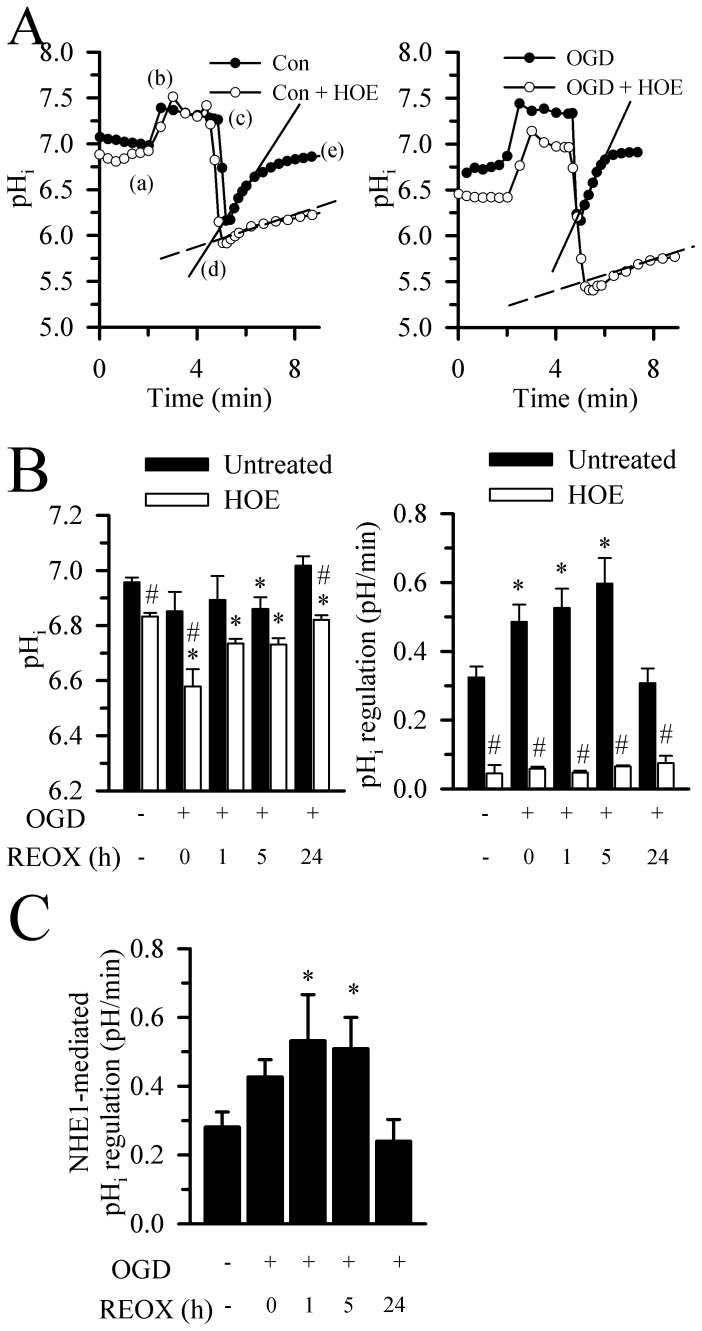
Increased NHE1 activity in hippocampal astrocytes following OGD/REOX. **A.** pH_i_ changes were determined by BCECF-AM dye in astrocytes subjected to NH_4_^+^/NH_3_ prepulse-mediated acid-loading. Either normoxic astrocytes (left panel) or astrocytes at 2 h OGD (right panel) were exposed to 30 mM NH_4_Cl_3_ (**a–c**), then returned to standard HCO_3_^–^free HEPES-MEM solution (**c–e**). After an initial acidification, pH_i_ recovery followed (**d–e**). The pH recovery rates were determined at ∼ 6.5 to normalize for the allosteric regulation of H^+^ on NHE1 activity. A slope of the pH_ i_ changes following the prepulse was calculated as pH_i_ recovery rate (solid or dashed line). For HOE 642 treatment, the drug was present throughout the experiment. **B.** pH_i_ (left panel) and pH_i_ recovery rates (right panel) were summarized under normoxic control, 2 h OGD, or 1, 5, or 24 h REOX conditions. **C.** Summary data of NHE1-mediated recovery rates under normoxic and OGD/REOX conditions. The values of ∼20 cells from each coverslip/culture were averaged. The n values were the number of cultures under each condition and indicated as normoxia (9), normoxia+HOE 642 (6), 0 REOX (5), 0 REOX+HOE 642 (4), 1 h REOX (3), 1 h REOX+HOE 642 (4), 5 h REOX (5), 5 h REOX+HOE 642 (4), 24 h REOX (4) and 24 h REOX+HOE 642 (3). Data are expressed as mean ± SEM. *p<0.05 vs. corresponding untreated. # p<0.05 vs. corresponding untreated.

We then investigated changes of NHE1 activity by measuring basal pH_i_ and pH_i_ recovery rate in these astrocytes in response to an NH_3_/NH_4_^+^ prepulse-induced acidification. As shown in **Figure 2** **A (left panel)**, when normoxic control astrocytes were exposed to 30 mM NH_3_/NH_4_^+^, pH_i_ rose rapidly as NH_3_ diffused into the cell and combined with H^+^ to form NH_4_^+^ (**a–b**) and then declined slowly over 2.5 min (**b–c**). Returning cells to standard HCO_3_^–^free HEPES-MEM solution caused pH_i_ to decrease due to the rapid efflux of NH_3_ from cells and trapping H^+^ inside the cells (**c–d**). Normoxic control cells were able to restore pH_i_ to their basal levels over time (**Figure 2** **A, d–e**). However, when NHE1 protein was inhibited with its potent inhibitor HOE 642 (1 µM), cells nearly failed to recover the pH_i_. This suggests that under normal HCO_3_^–^free conditions, hippocampal astrocytes largely depend upon NHE1 activity to regulate pH_i_.

The OGD-treated reactive astrocytes exhibited a more acidic basal pH_i_ compared to control cells (6.74±0.03 vs. 6.95±0.02 pH units, p<0.05, **Figure 2** **A, B**). A faster pH_i_ recovery with a steeper slope was detected following the NH_3_/NH_4_^+^ prepulse-induced acidification in the OGD-treated reactive astrocytes (**Figure 2** **A, right panel and** **Figure 2** **B, right panel**). This elevated H^+^ extrusion in the OGD-treated cells was mediated by NHE1 activity because blocking NHE1 activity with HOE 642 nearly abolished the pH_i_ recovery (**Figure 2A, right panel**). Moreover, hippocampal astrocytes maintained a relatively stable pH_i_ during 1–24 h REOX **(** **Figure 2** **B, left panel**). At 5 h REOX, the pH_i_ was reduced from 6.96±0.02 to 6.86±0.04. By 24 h REOX, the astrocytes became slightly alkaline (7.02±0.03). Interestingly, inhibition of NHE1 with HOE 642 acidified astrocyte pH_i_ under both normoxic conditions and 2 h OGD or 1–24 h REOX **(** **Figure 2** **B, left panel).** The strongest effect of NHE1 blockade occurred at 2 h OGD. On the other hand, 2 h OGD increased pH_i_ regulation rate from a basal level of 0.32±0.03 to 0.53±0.06 pH unit/min (p<0.05). This elevation on H^+^ extrusion was sustained through 1–5 h REOX. At 24 h REOX, the pH_i_ regulation rate returned to the basal level. In the presence of NHE1 inhibitor HOE 642, hippocampal astrocytes failed to regulate pH_i_ under all conditions (∼ 0.05 pH unit/min). This suggests that the OGD/REOX caused a sustained increase in H^+^ efflux, which is largely via stimulation of NHE1 activity. This view was further demonstrated in **Figure 2** **C.** HOE 642-sensitive pH_i_ recovery rate (NHE1-mediated) was increased at 1–5 h REOX. At 24 h REOX, NHE1-mediated pH_i_ regulation rate returned to control values. These data suggest that NHE1 plays an important role in maintaining pH_i_ in hippocampal astrocytes and its stimulation contributes to H^+^ extrusion and intracellular alkalization after OGD/REOX.

### Changes of [Na^+^]_i_ and [Ca^2+^]_i_ in Hippocampal Astrocytes Following OGD/REOX

**Figure 3:**
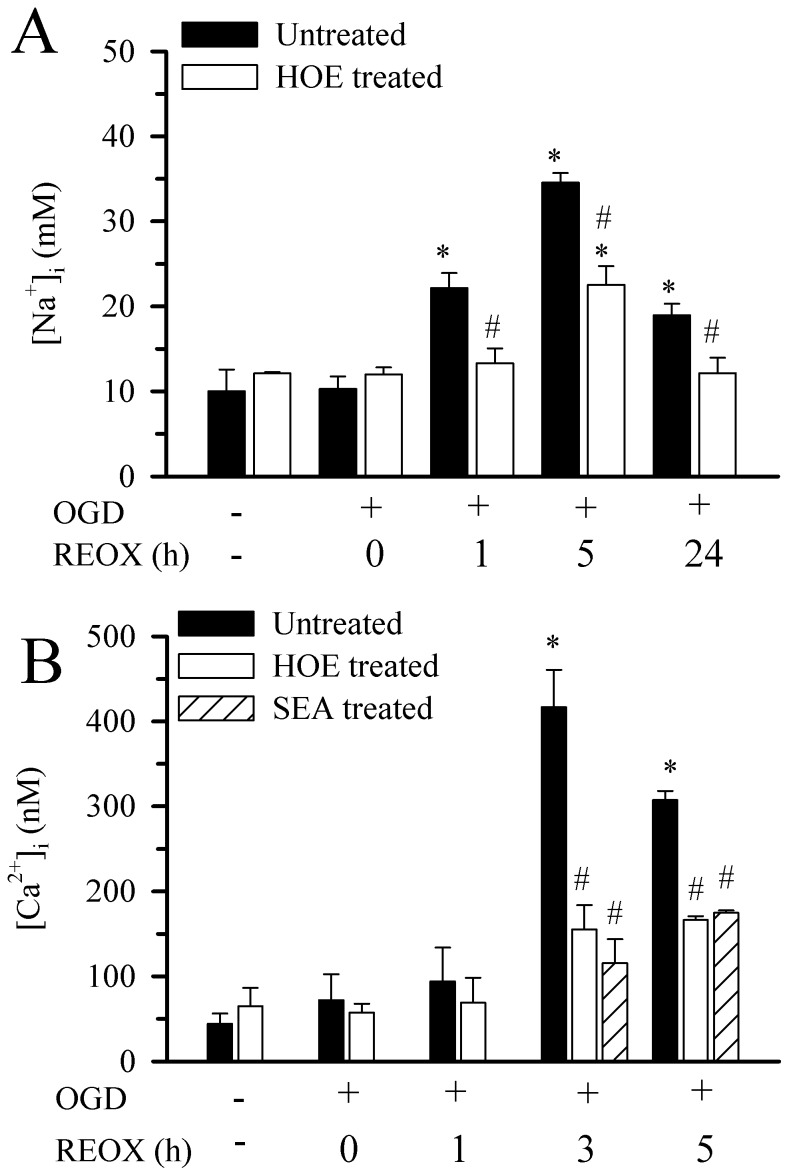
Changes of [Na^+^]_i_ and [Ca^2+^]_i_ in hippocampal astrocytes following OGD/REOX. **A.** [Na^+^]_i_ in hippocampal astrocyte cultures was determined with Sodium Green dye at 2 h normoxia or after 2 h OGD followed by 0, 1, 5, or 24 h REOX. HOE 642 (1 µM) was present during REOX only. Data are mean ± SEM. The values of ∼10 cells from each coverslip/culture were averaged. N of 3 cultures were for all groups except for normoxia, 0 h REOX, and 24 h REOX+HOE 642 (n = 4) or.5 h REOX and 24 h REOX (n = 5). *p<0.05 vs. normoxic control, # p<0.05 vs. corresponding untreated. **B.** [Ca^2+^]_i_ in hippocampal astrocytes following OGD/REOX. [Ca^2+^]_i_ in hippocampal astrocyte cultures was determined at 2 h normoxia or after 2 h OGD followed by 0, 1, 3, or 5 h REOX using fura-2 AM. HOE 642 (1 µM) or SEA 0400 (1 µM) was present during REOX only. The values of ∼10 cells from each coverslip/culture were averaged. Data are expressed as mean ± SEM. N of 4 cultures were for normoxia and the rest of other conditions were n of 3 cultures. *p<0.05 vs. normoxic control, # p<0.05 vs. corresponding untreated.

We investigated whether overstimulation of NHE1 results in Na^+^ and Ca^2+^ overload in reactive astrocytes after OGD/REOX. First, we determined [Na^+^]_i_ in hippocampal astrocytes during OGD/REOX. The resting level of [Na^+^]_i_ in hippocampal astrocytes was 10.0±2.6 mM and did not change significantly when NHE1 was inhibited (12.1±0.1 mM). Following 2 h OGD, [Na^+^]_i_ was not different from normoxia (**Figure 3** **A**). Upon REOX, [Na^+^]_i_ increased to 22.1±1.8 mM at 1 h REOX and 34.5±1.1 mM by 5 h of REOX (p<0.05, **Figure 3** **A**). At 24 h of REOX, [Na^+^]_i_ decreased by 45% but remained significantly elevated compared to normoxic control (18.9±1.4 mM). In contrast, inhibition of NHE1 activity with 1 µM HOE 642 reduced [Na^+^]_i_ by ∼ 75% (13.3±1.8 vs. 22.1±1.8 mM) at 1 h REOX and ∼ 50% (22.5±2 mM vs. 34.1±1 mM) at 5 h REOX **(** **Figure 3** **A)**. After 24 h REOX, [Na^+^]_i_ in the HOE-treated cells returned to normoxic levels (12.1±1.8 mM). Taken together, these data indicate that activation of NHE1 activity led to sustained Na^+^_i_ overload in reactive astrocytes after OGD/REOX.

We further investigated whether Na^+^_i_ overload could induce an elevation in [Ca^2+^]_i_ via reversal of Na^+^/Ca^2+^ exchanger (NCX_rev_) in hippocampal astrocytes following OGD/REOX. Basal [Ca^2+^]_i_ in hippocampal astrocytes was 44.2±12.1 nM and was not affected by 1 µM of HOE 642 (65.2±21.2 nM). [Ca^2+^]_i_ remained unchanged following 2 h OGD and at1 h REOX **(** **Figure 3** **B)**. However, at 3 h REOX, [Ca^2+^]_i_ in astrocytes increased significantly (416.7±43.9 nM), and remained elevated at 5 h of REOX (307.4±10.6 nM, p<0.05, **Figure 3** **B**). Interestingly, in hippocampal astrocytes treated with 1 µM HOE 642 the increase in [Ca^2+^]_i_ was much less at either 3 h REOX (155.2±28.6 nM) or at 5 h REOX (166.3±4.5 nM, **Figure 3** **B**). This implies that the rise in [Ca^2+^]_i_ may result from increased NCX_rev_ subsequent to Na^+^_i_ overload (although we did not determine [Na^+^]_i_ at 3 h REOX, it remained elevated at 5 h REOX and could stimulate NCX_rev_). To test this possibility, we examined whether SEA 0400 (1 µM), a potent inhibitor of NCX_rev_, could block the increases in Ca^2+^ after OGD/REOX in hippocampal astrocytes. As shown in **Figure 3** **B,** inhibition of NCX_rev_ with SEA 0400 abolished the OGD/REOX-mediated rise in [Ca^2+^]_i_. Interestingly, SEA 0400 effects are similar to the inhibition of NHE1 by HOE 642. These data illustrate that NHE1 activation in hippocampal astrocytes can couple with reverse mode operation of NCX_rev_ and result in Ca^2+^ elevation subsequent to intracellular Na^+^ overload.

### Roles of NHE1 in Glutamate and Cytokine Release from Hippocampal Astrocytes following OGD/REOX

**Figure 4:**
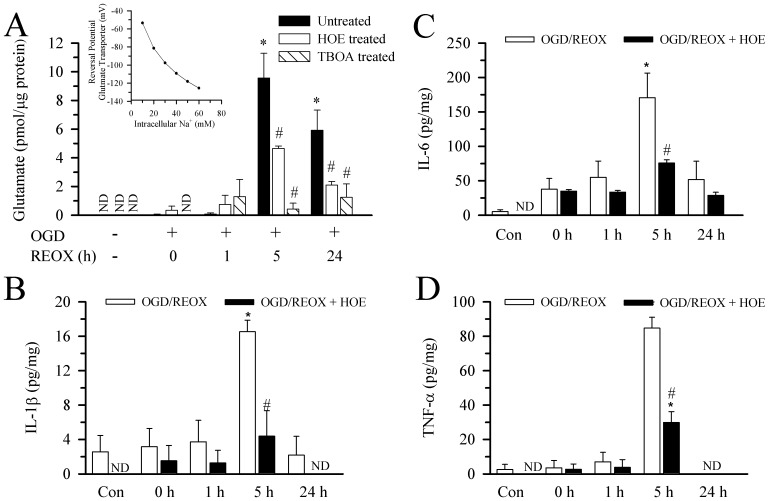
Glutamate and pro-inflammatory cytokine release from hippocampal astrocytes following OGD/REOX. **A.** Glutamate release in hippocampal astrocyte cultures was determined at 2(1 µM) or TBOA (100 µM) was present during REOX only. Data are mean ± SEM. N of 4 cultures were for all groups except for normoxia (n = 5). *p<0.05 vs. normoxic control, # p<0.05 vs. corresponding untreated. ND: not detectable. **Inset:** The reversal potential for the glutamate transport was plotted as a function of [Na^+^]_i_. The known values for [Na]_o_, [H]_o_, [H]_i_ and [K]_o_ at baselines were used along with an assumed value for [Glu]_o_ of 0.01 µM, [Glu]_i_ of 5 mM and [K]_i_ of 70 mM. **B–D**. Release of innate immune cytokines in the culture medium of hippocampal astrocytes. HOE 642 (1 µM) was present during normoxia or REOX treatment. IL-1β (**B**) IL-6 (**C**), or TNF-α (**D**) were normalized to cell lysate protein and expressed as pg/mg protein. Data are mean ± SEM (n = 3). ND: not detectable. *p<0.05 vs. normoxic control. # p<0.05 vs. corresponding untreated.

We speculate that overstimulation of NHE1 activity may affect glutamate release and/or uptake via disruption of Na^+^ and H^+^ homeostasis. To test this, we measured glutamate release from astrocytes after OGD/REOX. No detectable glutamate release was found in medium of the normoxic hippocampal astrocyte cultures (**Figure 4** **A**). Following 2 h of OGD or 1 h REOX, low levels of glutamate (0.04±0.04 pmol/µg protein) were released from hippocampal astrocytes. These data suggest that hippocampal astrocytes release minimal amount of glutamate after OGD or early REOX. In contrast, following 5 h REOX, glutamate release from hippocampal astrocytes increased dramatically to 9.6±1.7 pmol/µg protein (**Figure 4** **A)**. At 24 h REOX, although the glutamate levels in the media were reduced, they remained elevated compared to normoxic levels (5.9±1.4 pmol/µg protein, **Figure 4** **A**). Inhibition of NHE1 activity in hippocampal astrocytes with 1 µM HOE 642 had no effect on the release of glutamate at 2 h OGD or 1 h REOX (p>0.05, **Figure 4** **A**). However, blocking NHE1 activity decreased the glutamate release by ∼ 50% at 5 h and ∼ 35% at 24 h REOX (**Figure 4** **A**). It is unclear how blocking NHE1 activity can reduce glutamate release from astrocytes following OGD/REOX.

To test whether glutamate release was mediated via reversal of Na^+^-dependent excitatory amino acid transporter (EAATs) subsequent to increases in Na^+^_i_ **,** we investigated if the competitive non-transportable EAAT blocker TBOA can block the glutamate release. 100 µM TBOA had no effects on the glutamate release at 2 h OGD or 1 h REOX (p>0.05, **Figure 4** **A**). The lack of effect on glutamate release further implies that little glutamate is released during this initial OGD/REOX period. However, TBOA nearly abolished the glutamate efflux (∼95%) at 5 h REOX and reduced the glutamate release by 80% at 24 h REOX. These data further suggest that glutamate release during REOX was primarily through reversal of EAATs. To further confirm glutamate release through reversal of the transporter, we calculated the reversal potential for the transporters using the following equation.
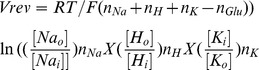
Where R is the gas constant, T is temperature, F is the Faraday constant, and V*_rev_* is the reversal potential. The values for [Na]_o_, [Na]_i_, [H]_o_, [H]_i_ and [K]_o_ are known, the [Glu]_o_ were estimated to be 0.01 µM at baseline, 0.1 µM at 5 h REOX and 0.05 µM at 24 h REOX. Assuming a value for [Glu]_i_ of 5 mM and [K]_i_ of 70 mM, then V*_rev_* = −53.6 mV. Cultured cortical astrocytes have a large range of resting plasma membrane potentials, but average ∼ −60 mV when measured either by whole cell or perforated electrophysiological recordings [16]. Thus, under culture conditions, when the [Glu]_o_ was very low, the glutamate transporters were near their reversal potentials and little or no flux through the transporter would be expected. In contrast, because of the stoichiometry of three Na^+^/glutamate anion transporter, the reversal potential was sensitive to changes of [Na]_i_ (**Figure 4** **A inset**). At 5 h REOX with a [Na]_i_ of 34.5 mM and [K]_i_ of 45.5 mM, the reversal potential dropped to −78.2 mV and efflux of glutamate through the transporters would be favored. At 24 h REOX, when [Na]_i_ decreased to 19 mM the reversal potential fell to −59.6 mV, making glutamate release less likely.

Next, we accessed the release of pro-inflammatory cytokines IL-1β, IL-6 and TNF-α from hippocampal astrocytes following OGD/REOX. Neither OGD nor 1 h REOX resulted in significant release of the cytokines (**Figure 4** **B–D**). However, after 5 h REOX, we detected a transient increase in the release of IL-1β, IL-6 and TNF-α by 6-fold, 33-fold and 33-fold, respectively. Similar to the glutamate release, the release of the proinflammatory cytokines from hippocampal astrocytes returned to control levels by 24 h REOX (**Figure 4** **B–C**). Interestingly, in the presence of the NHE1 inhibitor HOE 642 (1 α µM), the release of cytokines at 5 h REOX was reduced between 26% and 45% (p<0.05). These data imply that inhibition of NHE1 activity also decreases cytokine release from reactive astrocytes.

**Figure 5:**
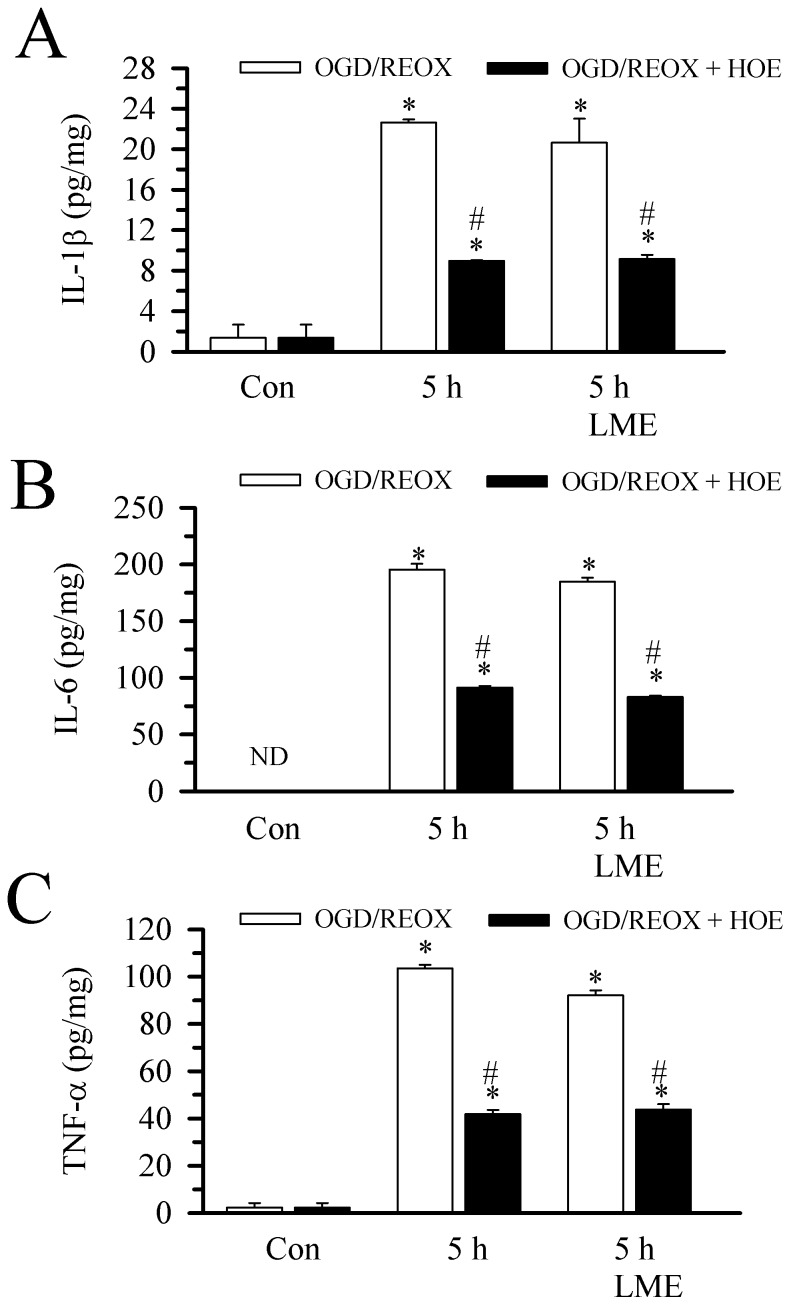
Pro-inflammatory cytokine release from LME-treated hippocampal astrocyte cultures following OGD/REOX. Release of innate immune cytokines was detected in the culture medium of hippocampal astrocytes at 2(1 µM) was present during normoxia or REOX treatment (with 5 mM LME). IL-1β (**A**), IL-6 (**B**), TNF-α (**C**). Cytokine content was normalized to cell lysate protein and expressed as pg/mg protein. Data are mean ± SEM (n = 3). *p<0.05 vs. normoxic control. # p<0.05 vs. corresponding untreated, ND: not detectable.

To rule out that possible contaminating microglia in the astrocyte culture contribute to the cytokine release in the study, we measured the cytokine release from LME-treated astrocyte cultures which were depleted of microglia (**Figure 5**). In the microglia-depleted astrocyte cultures, similar levels of cytokine release were detected from the astrocyte cultures at 5 h REOX. Inhibition of NHE1 significantly reduced the cytokine release. Taken together, these findings firmly suggest that activation of NHE1 during 5 h REOX not only triggered glutamate release, but also increased pro-inflammatory cytokine release in hippocampal astrocytes.

### Minimal Cell Death of Reactive Astrocytes After OGD/REOX

**Figure 6:**
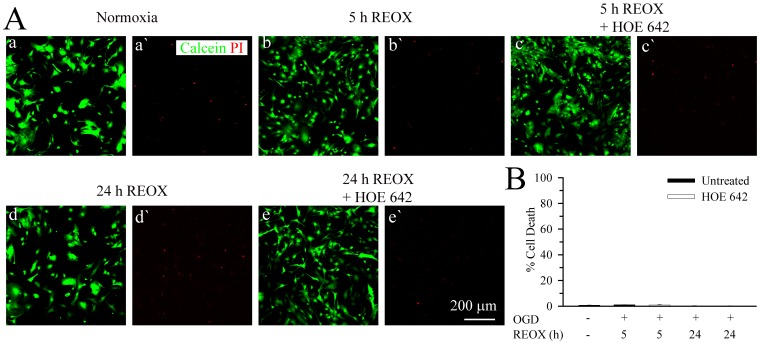
Minimal cell death of reactive astrocytes after OGD/REOX. Hippocampal astrocytes were grown on 24 well culture dishes and subjected to 2(1 µM) was present only during REOX. At the end of REOX, cells were loaded with calcein-AM (green) and PI (red). **A**. Representative images of live (calcein positive) and dead (PI positive) cells of normoxia (a, à), 5 h REOX (b, b`), 5 h REOX+HOE 642 (c, c`), 24 REOX (d, d`) or 24 REOX+HOE 642 (e, è). **B**. Summary of reactive astrocyte death from six wells from each experimental condition. Data are mean ± SEM. n = 6.

We further investigated the impact of the above cellular changes on astrocyte viability. Under normoxic conditions, astrocytes in the culture retained the calcein dye with minimum PI staining (**Figure 6A** **a, à**). The cell death rate was 0.83±0.14% (**Figure 6** **B**). When astrocytes were subjected to OGD, followed with either 5 h REOX (**Figure 6** **A b, b`**) or 24 REOX (**Figure 6** **A d, d`),** the cell death rates remained at ∼1% (**Figure 6** **B**). Inhibition of NHE1 with HOE 642 did not change the cell death in astrocytes (**Figure 6** **A c, c` and** **Figure 6** **A e,è**). These data suggest that reactive astrocytes were resistant to ischemia-mediated cell death.

## Discussion

### Role of NHE1 in Regulation of pH_i_ in Hippocampal Astrocytes Following OGD/REOX

In this study, the resting pH_i_ in murine hippocampal astrocytes was 6.94±0.02 under HCO_3_^–^free normoxic conditions. This is consistent with reports of resting pH_i_ values under HCO_3_^−^ -free conditions that range from 6.88 to 7.05 in rat hippocampal astrocytes [17]–[19]. Bevensee et al. (1997) reported that under nominally HCO_3_^−^ free conditions, 88% of the prepulse-induced pH_i_ recovery rate in rat hippocampal cultures was sensitive to 0.9 mM amiloride [20]. In addition, when amiloride was applied under steady state conditions, there was a decrease in pH_i_. Similar results were reported with 1 mM amiloride [21]. In the current study, using the potent NHE1 inhibitor HOE 642 (1 µM), we observed 86% blockade of the pH_i_ recovery rates in hippocampal astrocytes. Thus, in the absence of HCO_3_^−^, NHE1 plays a dominant role in pH_i_ regulation in hippocampal astrocytes. Indeed, mRNA expression for NHE1 in brain is ten-times the level of other NHE isoforms [22]. NHE2 and NHE3 transcripts were only present in the granule and Purkinje cells of the cerebellum. NHE4 protein expression is undetectable in hippocampal astrocyte cultures [18]. On the other hand, there is weak expression of NHE3 protein in mouse hippocampus [23]. The other plasma membrane NHE5 has been characterized as largely insensitive to amiloride, and is restricted to the cell bodies of neurons of the hippocampus [24], [25]. Taken together, NHE1 is the primary NHE isoform in regulation of pH_i_ in hippocampal astrocytes and the contribution of other NHE isoforms is minimal. The residual NHE1-independent pH_i_ regulation likely results from activity of a H^+^ pump [21] or lactate-H^+^ co-transport [26].

In the current study, 2 h of OGD led to an increase in NHE1 expression in hippocampal astrocytes that was sustained during 5 h REOX. The underlying mechanisms for this upregulation are unknown. Previously, we observed an increase in HIF-1α expression in cortical neurons that preceded the up-regulation of NHE1 protein after OGD/REOX [27]. Given that the promoter region of NHE1 gene contains a candidate-binding site for HIF-1α it is likely that HIF-1α plays a role in hypoxia-induced up-regulation of NHE1 [28].

### NHE1 Activation and Glutamate Release from Hippocampal Astrocytes After OGD/REOX

Rapid removal of glutamate from extracellular space is primarily a function of astrocytes and is critical for neuronal physiology and survival. However, under both physiological and pathophysiological conditions, astrocytes will release glutamate through a number of mechanisms including swelling-induced anion channel opening and ionotropic purinergic receptors, Ca^2+^- dependent exocytosis, or glutamate exchange via the cysteine-glutamate antiporter [29]. However, reversal of Na^+^-dependent glutamate transporters has been implicated as a major factor responsible for excessive glutamate release during ischemia that leads to a cytotoxic cascade and neuronal death [29]. To date, five different high-affinity glutamate transporters have been cloned: GLAST, GLT, EAAC, EAAT4 and EAAT5. However, GLT-1 and GLAST are together responsible for most of the glutamate uptake activity in most regions of the mammalian CNS and are only found in astroglia in normal mature brains [30]. This highlights the importance of astroglia in extracellular glutamate removal [30]. Developmentally, GLAST expression dominates early in astrocytes but is overtaken by GLT-1 expression with maturity. In primary rat astrocyte cultures, it has been shown that only GLAST is expressed unless the cells are treated with dBcAMP or grown in the presence of neurons [31]. Under these conditions, GLAST expression was increased by two-three fold and GLT-1 expression was robustly induced. Similar results have been shown in primary hippocampal astrocytes grown with neurons [32]. Thus, in our differentiated cultures, we would expect both isoforms are involved (data not shown).

In vivo, it has been estimated that glutamate uptake is made against an extracellular to intracellular gradient of ∼ 1∶2500 [33]. To accomplish this, glutamate transport is coupled to the transport of Na^+^ and K^+^ down their respective concentration gradients. In addition, because of the stoichiometry of three Na^+^ to one H^+^ and the counter-transport of one K^+^ for each glutamate anion moved into the cell, there is a net movement of two positive charges and the plasma membrane potential likely contributes to the driving force [34], [35]. Therefore, changes of Na^+^ and H^+^ homeostasis can alter the driving force of glutamate uptake via EAATs and trigger its release from astrocytes.

In this study, we detected concurrent intracellular Na^+^ overload and glutamate release from hippocampal astrocytes after 5 h of REOX following 2 h of OGD. This release was largely inhibited by TBOA, a nontransportable inhibitor of GLAST and GLT-1, suggesting that the glutamate release was the result of glutamate transport reversal. Interestingly, in the presence of HOE 642, the calculated reversal potential of the glutamate transported at 5 h REOX was −57.94 mV (vs. −78.2 mV at OGD/REOX), suggesting that inhibition of NHE1 and subsequent reduction of Na^+^ overload would help to prevent EEATs-mediated glutamate release from astrocytes following ischemia. These findings are consistent with other previous reports. Investigators have reported neuroprotection with the use of glutamate transporter inhibitors during bilateral carotid occlusion/hypotension in rats [36], neonatal hypoxic ischemia [37], and in rat dorsal column slices exposed to anoxia [38]. EEAT reversal in astrocytes has been demonstrated *in vitro* by raising extracellular K^+^ [39], [40], OGD perfusion [41], or metabolic blockade using idoacetate/KCN or NaF/azide [42], [43].

Xie et al measured changes in astrocytic plasma membrane potential when hippocampal slices were exposed to OGD [44]. They found that 30 min of OGD caused membrane potential to depolarize by ∼ 25 mV, but when the slice was reoxygenated there was a transient hyperpolarization of the plasma membrane by 9 mV before returning to the pre-OGD potential. This hyperpolarization was attributed to enhanced Na^+^/K^+^-ATPase activity because it could be blocked by the application of ouabain [45]. We have previously reported that 2 h OGD in cultured cortical astrocytes results in an ∼ 30% decrease in ATP content [13]. Thus, while OGD results in a partial loss of plasma membrane potential in hippocampal astrocytes, Na^+^/K^+^-ATPase activity appears to still be functional. Therefore, increased Na^+^ influx in hippocampal astrocytes after OGD/REOX, rather than altered Na^+^ pump function or membrane depolarization, seem to drive reversal of EAATs in hippocampal astrocytes.

### NHE1 Activation and Cytokine Release from Reactive Astrocytes After OGD/REOX

It is well recognized that the activation of astrocytes is accompanied by increased production of potentially neurotoxic factors, including cytokines [46] Both astrocytes and microglia have been shown to release a myriad of pro- and anti- inflammatory cytokines, including interleukins, interferons and tumor necrosis factors, as well as chemokines [47]. Findings from Lau and Yu (2001) suggest that astrocytes respond to pathophysiological conditions by releasing cytokines [48]. It has been proposed that a central role of astrocytes in regulating neuroinflammation in the CNS is via the NF-κB pathway [49], [50]. Here we observed that 5 h REOX significantly increased the secretion of several inflammatory cytokines (IL-1β, IL-6 and TNF-α) in astrocytes. Interestingly, the release was ablated by the inhibition of NHE1. Increases in intracellular Na^+^ and Ca^2+^ levels in hippocampal astrocytes after OGD/REOX may be the trigger behind astrocytic activation and subsequent cytokine release. Collectively, these results suggest that astrocytes-induced cytokines release following *in vitro* HI may be regulated by NHE1 and changes of Na^+^ and Ca^2+^.

In a recent interesting study, Kim et al. (2011) demonstrated a role for intracellular Ca^2+^ as a signal pathway for astrocyte mediated cytokines release [51]. The authors reported that cytokines produced by primary astrocytes and bone marrow-derived mast cell co-cultures are mainly induced by signaling via Ca^2+^/PKCs/MAP kinases/NF-κB/STAT^1727^ pathways and the cytokines produced subsequently re-activate astrocytes via Jak/STAT1^701^, which then release more cytokines thereby contributing to the exacerbation of experimental allergic encephalomyelitis in mice. The authors reported that activation of astrocytes is usually manifested as a rise of intracellular Ca^2+^ level due to release of Ca^2+^ from internal stores as well as Ca^2+^ uptake from the extracellular space. This suggests that NHE1 may have an important role in astrocyte mediated inflammatory responses following HI. Recently, we have shown that NHE1 is required for microglial activation and proinflammatory responses after ischemia. In cultured microglia, activation by several stimuli depends on NHE1 mediated H^+^ homeostasis and inhibition of NHE1 with HOE 642 also reduced the production of superoxide anions as well as proinflammatory cytokines IL-1β, IL-6, and TNF-α induced by LPS or *in vitro* ischemia [52]. These results were supported by evidence from *in vivo* ischemia studies where activation of microglia leading to proinflammatory cytokine production and phagocytosis is significantly reduced with NHE1 inhibition or genetic knockdown [53].

**Figure 7:**
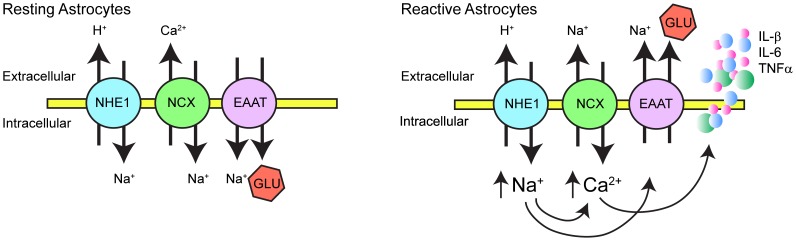
Proposed role of NHE1 in reactive hippocampal astrocytes following OGD/REOX. In resting astrocytes under normoxic conditions, NHE1 and NCX function in removal of intracellular H^+^ and Ca^2+^. Glutamate transporters (EAAT) uptake glutamate (Glu) into astrocytes to maintain its low extracellular content. In response to OGD/REOX, up-regulation of NHE1 promotes sustained H^+^ efflux in exchange of Na^+^ influx in reactive astrocytes. This Na^+^ overload triggers the reversal mode operation of NCX and Ca^2+^ influx. The intracellular Ca^2+^ rise serves as a signal to release innate cytokines, while elevation of Na^+^_i_ leads to Glu release either via decreased uptake of Glu through EEAT or their reversed operation.

In summary, NHE1 protein expression was up-regulated in reactive hippocampal astrocytes after *in vitro* ischemia (**Figure 7**). Concurrently, NHE1 activity is elevated, [Na^+^_i_] and [Ca^2+^_i_] increased, leading to cytokine and glutamate release from hippocampal astrocytes but did not cause cell death. Interestingly, although inhibition of NHE1 activity did not change reactive astrocyte death, it significantly reduced the release of glutamate and cytokines. Therefore, we concluded that over-stimulation of NHE1 promotes gliotransmitter and cytokine release from reactive astrocytes, which can subsequently contribute to hippocampal neuronal damage under hypoxic and ischemic conditions.
